# Porcine model for the study of liver regeneration enhanced by non-invasive ^13^C-methacetin breath test (LiMAx test) and permanent portal venous access

**DOI:** 10.1371/journal.pone.0217488

**Published:** 2019-05-31

**Authors:** Eva-Maria Wittauer, Felix Oldhafer, Eva Augstein, Oliver Beetz, Moritz Kleine, Carsten Schumacher, Lion Sieg, Hendrik Eismann, Kai Johanning, André Bleich, Florian Wolfgang Rudolf Vondran

**Affiliations:** 1 ReMediES, Department of General, Visceral and Transplant Surgery, Hannover Medical School, Hannover, Germany; 2 Department of Anaesthesiology and Intensive Care Medicine, Hannover Medical School, Hannover, Germany; 3 Institute for Laboratory Animal Science, Hannover Medical School, Hannover, Germany; University of Nebraska Medical Center, UNITED STATES

## Abstract

**Introduction:**

Despite advances in perioperative management and surgical technique, postoperative liver failure remains a feared complication after hepatic resection. Various supportive treatment options are under current discussion, but lack of structured evaluation. We therefore established a porcine model of major liver resection to study regeneration after partial hepatectomy in a reliable and well-defined pre-clinical setting.

**Methods:**

Major hepatectomy was performed on seven minipigs with the intention to set up a non-lethal but relevant transient impairment of liver function. For steady postoperative vascular access (e.g. for blood withdrawal, measurement of venous pressure), permanent catheters were implanted into the internal jugular and portal veins, respectively. Animals were followed up for 30 days; clinical and laboratory results were recorded in detail. Monitoring was enhanced by non-invasive determination of the maximum liver function capacity (LiMAx test).

**Results and conclusions:**

The established porcine model appeared suitable for evaluation of postoperative liver regeneration. Clinical characteristics and progression of liver function impairment as well as subsequent recovery were comparable to courses known from surgery in humans. Laboratory parameters (e.g. liver enzymes, bilirubin, INR, coagulation factor II) showed relevant derangements during postoperative days (POD) 0 to 3 followed by normalization until POD 7. Application of the LiMAx test was feasible in minipigs, again showing values comparable to humans and kinetics in line with obtained laboratory parameters. The exteriorized portal vein catheters enabled intra- and postoperative monitoring of portal venous pressures as well as easy access for blood withdrawal without relevant risk of postoperative complications.

## Introduction

In the last decades, advances in perioperative management and surgical techniques improved the safety and extent of liver resections [1]. Functional and imaging measures to predict liver remnant function as well as preventive interventions such as portal vein embolization or the Associating Liver Partition and Portal Vein Ligation for Staged Hepatectomy (ALPPS) procedure [2] were introduced. Nevertheless, post-hepatectomy liver failure (PHLF) remains a feared complication after liver resection with a high morbidity and mortality [2–6]. Basic treatment options of PHLF include common intensive care measures such as mechanical ventilation, dialysis, vasopressor therapy and administration of albumin and coagulation factors [5]. Specific therapeutic approaches include plasmapheresis, for which encouraging results have been reported in acute liver failure [7], and portal pressure modulation via various measures like portocaval anastomosis, ligation of the splenic artery and infusion of various vasoactive drugs that have already been applied in several experimental models [8–17]. The clinical relevance of these options though remains unclear. The only potential therapy with proven benefit for PHLF is salvage liver transplantation [18]. Nevertheless, this approach appears questionable due to possible delayed recovery of liver function, especially with current shortage of donor organs in mind. Furthermore, high post-transplantation mortality in the context of severe liver failure has been observed [18], and moreover transplantation often is impractical since patients with colorectal metastases, cholangiocarcinoma and hepatocellular carcinoma outside Milan criteria will not be considered. A promising alternative to bridge liver function until recovery could be hepatocyte transplantation [19]. Small case series or single case reports have already been published in context of acute liver failure (ALF) [20, 21], whereas comparable data is still lacking for PHLF.

The maximum liver function capacity (LiMAx) has recently been proposed as a novel ^13^C-breath test for the perioperative assessment of liver function [22]. This test is based on the metabolization of ^13^C-methacetin in the liver acinus [23] by liver-specific enzyme CYP1A2 into acetaminophen and carbon dioxide (^13^CO_2_). The latter than can be determined in the exhaled air, and a ratio of ^13^CO_2_/^12^CO_2_ is built to eventually calculate the maximum liver function capacity [24, 25]. In the last years, several studies have been published regarding the use of the LiMAx test to predict mortality and perioperative liver function or sepsis-related liver failure [26–30], but to our knowledge no data exist about its application in a pig model.

The underlying processes of liver regeneration thus need to be further investigated using a model with high degree of translation into humans and the opportunity for development and application of appropriate treatment strategies *in vivo*. Small animal models do not fulfill these criteria due to the lack of physiological comparability with humans—in contrast to pigs [31]. In rats for example liver resection of up to 90% is possible without development of fatal PHLF [32]. Nonetheless, large animal models are highly demanding regarding logistics, costs and time. Thus, aim of this study was to establish a porcine animal model to further study liver regeneration after major liver resection in a preclinical setting based on clinical, hemodynamic and conventional laboratory parameters as well as the novel non-invasive LiMAx test.

## Methods

### Legal approval

This study was performed at the Laboratory for Animal Science of Hannover Medical School after approval by the Lower Saxony regional authority for consumer protection and food safety (Niedersächsisches Landesamt für Verbraucherschutz und Lebensmittelsicherheit (LAVES); 16–2374). The animals were kept under housing conditions of the EU-Guideline 2010/63 and valid animal regulation act (Tierschutz-Versuchstierverordnung des deutschen Tierschutzgesetzes).

### Animals and preoperative care

**Fig 1:**
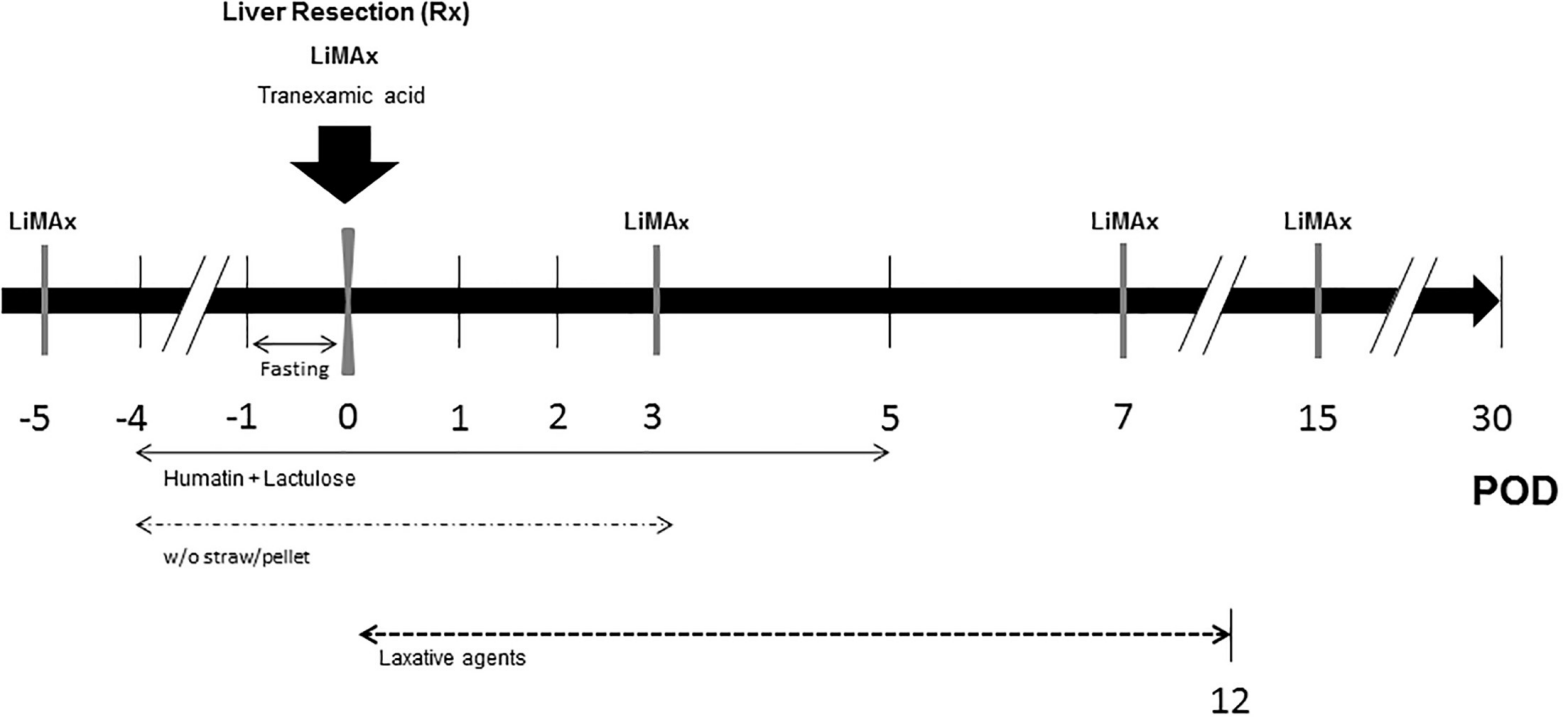
Simplified study design of the resection model. Maximum liver function capacity (LiMAx) was tested on day -5, 0 (after resection), 3, 7 and 15 (grey bars), respectively. Therapy with 1g paromomycin and 15ml lactulose-syrup twice a day was started 4 days prior to resection until day 5 postoperatively. Straw was removed from the compound from day -4 until day 3 and at the same time pigs were set on a pellet-free diet. Animals fasted 24 hours prior to surgery with free access to water. Further laxative agents applied: Macrogol 6.75g, paraffin oil 20ml and Microlax enema 5ml as needed. (POD: Postoperative day).

The study cohort consisted of 5 male and 2 female LEWE minipigs at the age of 524 ± 27 days with a weight of 49.9 ± 2.0 kg. The study design is shown in Fig 1. Initially, animals were bedded with straw and fed with pellets (Altromin Spezialfutter GmbH & Co. KG, Germany). Four days before surgery, bowel preparation was initiated to minimize the risk for hepatic encephalopathy and ileus after liver resection. Straw was replaced by a rubber mat and the animals were fed with liquid high-caloric food twice a day. Additionally, 1 g paromomycin per os and 15 ml of a lactulose-syrup were administered, orally. The animals fasted for 24 h prior to the onset of surgery with free access to water. In further 13 LEWE minipigs total hepatectomy was performed (following 24 hours of fasting with free access to water prior to surgery) in order to allow estimation of the weight of the future liver remnant (FLR) to define the degree of resection (see below).

### Anesthesia

Premedication consisted of intramuscular injection of 5 mg/kg tiletamine, 5 mg/kg zolazepam and 0.04–0.08 mg atropine. An ear vein was cannulated and anesthesia was induced with 1.5–2.5 mg/kg propofol intravenously. Endotracheal intubation was performed and subsequently controlled via auscultation and capnometry. Anesthesia was maintained with isoflurane at an expiratory concentration of 0.8–1.5 vol% and repetitive applications of 0.0025–0.007 mg/kg fentanyl i.v. according to clinical necessity. Before surgical incision, 1 g ampicillin, 0.5 g sulbactam and 0.5 g tranexamic acid were applied intravenously. Perioperative fluid and volume therapy consisted of an initial i.v. bolus of 15 ml/kg followed by 10 ml/kg/h of isotonic electrolyte solution. Perioperative monitoring of vital signs consisted of continuous pulse oximetry, ecg, non-invasive blood pressure measurement and capnography. During emergence from anesthesia, pigs received 4 mg/kg carprofen and 0.01 mg/kg buprenorphine i.v., respectively.

### Surgical procedures

#### Implantation of the central vein catheter

After shearing, sterile washing and covering of the throat and neck, a central venous catheter (customized 9F silicon catheter; SAI Infusion Technologies, USA) was placed into the internal jugular vein under sonographic guidance, applying the Seldinger technique and a peel-away sheath introducer (Li plus G, Biotronik, 10F). Correct positioning of the catheter was ascertained by intra-atrial ecg control (Alphacard B. Braun, Melsungen, Germany), X-ray imaging and/or the venous blood pressure profile. The hub of this silicon catheter was subcutaneously tunneled to the craniodorsal area of the right scapula.

#### Implantation of the exteriorized portal vein catheter

**Fig 2:**
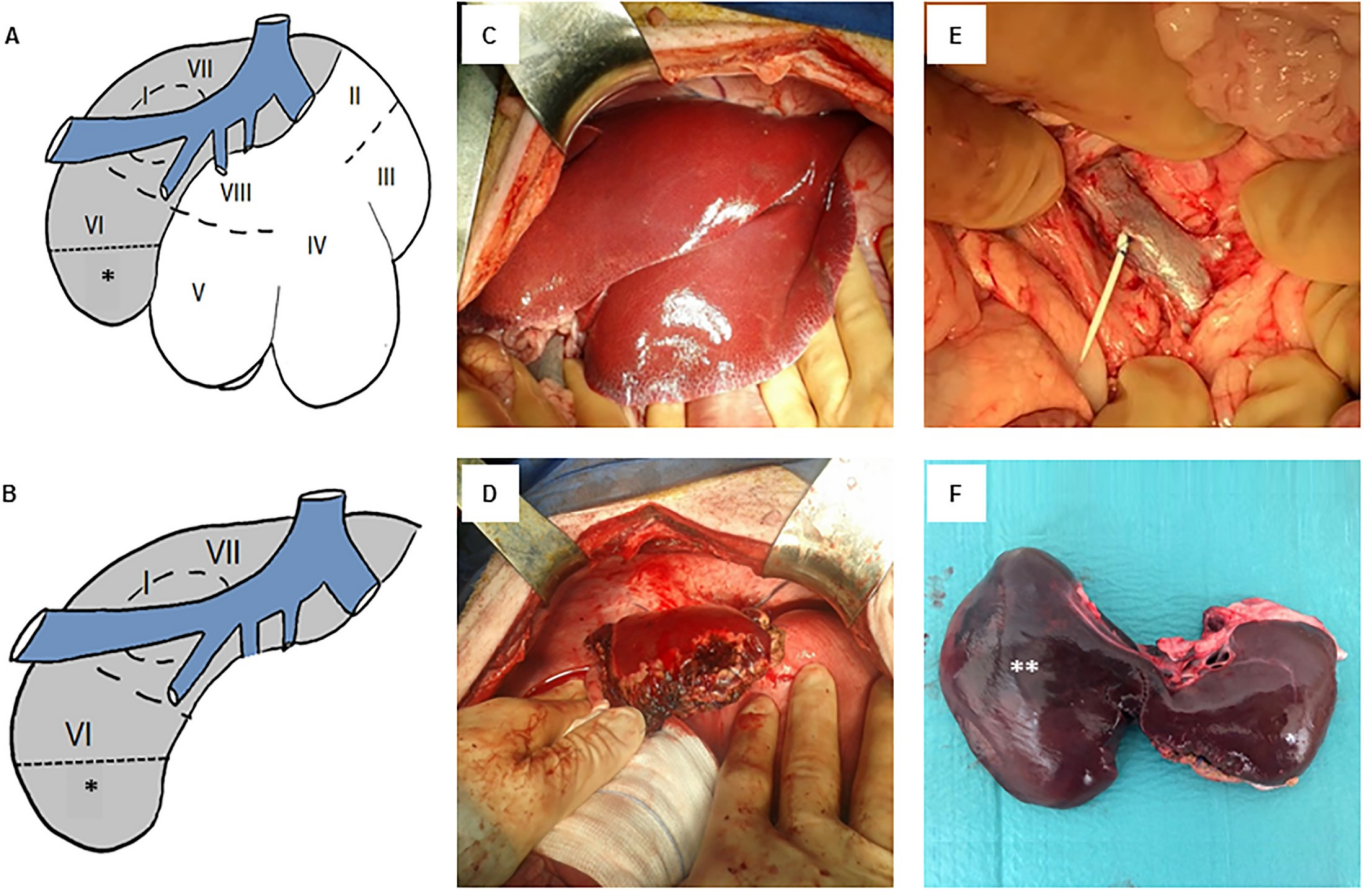
Portal venous catheter and liver resection. (**A**) Segmental anatomy of the porcine liver. Caudate lobe: Segment (I). Left lateral lobe: Segments (II) and (III). Left medial lobe: Segment (IV). Right medial lobe: Segments (V) and (VIII). Right lateral lobe: Segments (VI) and (VII). The white area represents the resected extend of three liver lobes and the grey area defines the future liver remnant (FLR); (*) representing the additional partial resection area of the right lateral liver lobe in order to achieve a FLR of approx. 50% is marked with an asterix (*). (**B**) Anatomy of the porcine following major liver resection, the asterix (*) again marks the potential additional resection area. (**C**) Anatomic situs prior to and (**D**) after major liver resection in the minipig. (**E**) Presentation of the superior mesenteric vein/portal vein following implantation of a 4–6 F tapered portal venous catheter. (**F**) Liver with hypertrophic right liver lobe (marked **) 30 days after liver resection.

After midline abdominal incision followed by intramuscular injection of 1–2 mg/kg lidocaine, the abdominal cavity was opened and explored. The pancreas was mobilized and the distal part of the portal vein was prepared, clamped with a Satinsky-clamp and the customized silicon catheter (4–6 F Tapered Catheter, SAI infusion technologies) was then inserted and secured (Fig 2E). The catheter was fixed to the peritoneum with sutures and tunneled trough the abdominal wall. Comparable to the central vein catheter, the final portal vein catheter hub position was located at the caudal side of the scapula.

#### Partial hepatectomy

**Fig 3:**
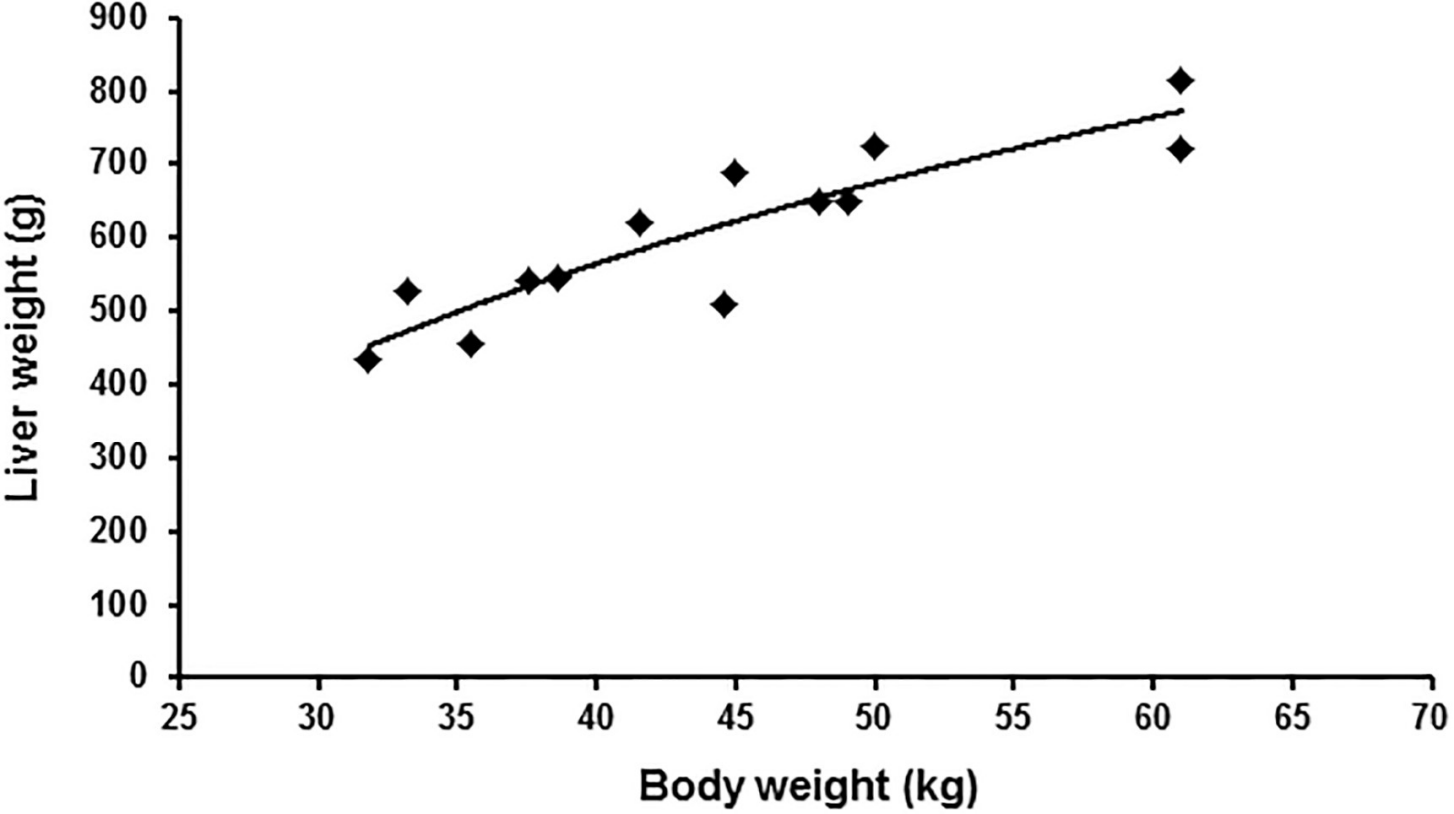
Calculation of the total liver weight. Diagram depicting total liver weight of 13 pigs following full hepatectomy in relation to bodyweight (black squares) as well as logistic regression curve (solid black line; LW = 491.86 *ln(BW)– 1249.6 (R^2^ = 0.807)) for estimation of the future liver remnant.

First, the liver was separated from the Ligamentum triangulare dextrum and falciform ligament. Subsequently, liver resection was performed either with a combination of vascular clamps and a monopolar knife or directly with a stapler (LC80 G, CHEX, Shanghai international Holding Corp. GmbH, Hamburg, Germany) depending on anatomical conditions of the individual liver lobes. After the resection, electrocoagulation and sutures with prolene 3–0 were performed until complete hemostasis. Partial hepatectomy was performed on three or four of five lobes (Fig 2A–2D), depending on individual anatomic conditions and size of the future liver remnant (FLR) aiming for 50% FLR based on total hepatectomies as calculated (Fig 3). Liver resection was accompanied by retrograde cholecystectomy. No abdominal drainages were introduced. Finally, closure of the abdomen and skin were performed by sutures. The pigs then were equipped with multifunctional jackets (worn 24 hours a day until euthanasia) to protect catheter hubs and to enable storage of mini infusion pumps for continuous postoperative infusion.

### Postoperative care

During the first postoperative 16 h, another 0.5 g tranexamic acid was given in 50 ml of 0.9% NaCl via a mini infusion pump (CRN CRONO Resérvoir, CANÈ MEDICAL TECHNOLOGY, CANÈ S.p.A., Italy). Over the first postoperative days, all pigs received adjusted fluid and volume therapy with isotonic electrolyte solution depending on respective levels of hemoglobin, hematocrit, lactate and metabolic status until values normalized (see below). Furthermore, animals received 40 mg pantoprazole twice a day in the first postoperative week and once a day in the second postoperative week. Additionally, the preoperative prophylaxis protocol for ileus and hepatic encephalopathy was repeated until day 5. On POD 1 to 3, 10–25 mg/kg/h metamizole and 0.01 mg/kg buprenorphine were given intravenously, respectively, twice a day. Additionally, 2 mg/kg carprofen was applied i.v. once a day. After POD 3 only metamizole was given on demand. Pigs were fed with high caloric liquid food for a minimum of two days. On POD 3 pellet nutrition was resumed in four or five portions per day. Once bowel function fully restored, pigs received full pellet nutrition. Animals were followed up for 30 days with planned euthanasia at the end of experiment (bolus injection of a lethal dose of pentobarbital sodium), clinical and laboratory results were recorded as described below.

### Conventional laboratory parameters

Animals were monitored via clinical chemistry (e.g. aspartate aminotransferase (AST), alanine aminotransferase (ALT), glutamate dehydrogenase (GLDH), γ-glutamyl transferase (GGT), alkaline phosphatase (AP), bilirubin, ammonia, creatinine, urea) and hematologic parameters (e.g. hemoglobin, hematocrit, thrombocytes, coagulation factor II, INR) using blood samples obtained via the central venous catheter. Ammonia additionally was monitored via the portal venous catheter, allowing for calculation of an ammonia clearance (portal venous level of ammonia minus central venous level of ammonia), before and 6 hours after liver resection.

All analyses were performed by the central laboratory of Hannover Medical School by standardized procedures. In general, blood samples were taken twice a day on POD 0 to 2 and once a day on POD 3 to 7 as well as on POD 11, 18, 21, 28 and prior to euthanasia, respectively. Perioperative, additional monitoring using conventional blood gas analyses (ABL 800 Flex, Radiometer GmbH, Krefled, Germany) was performed in order to control fluid management (e.g. lactate, hematocrit, hemoglobin).

### Non-invasive ^13^C-breath test (LiMAx test)

The maximum liver function capacity (LiMAx test) is based on indirect quantification of ^13^C-methacetin metabolism through expiration analysis of ^13^CO_2_/^12^CO_2_ ratio via infrared absorption spectroscopy method within the FLIP device (Flip 1.0; Humedics GmbH, Berlin, Germany) [33]. Exhalation of pigs was collected by a special cone placed on the spout and connected to the FLIP device. Each animal underwent LiMAx measurements at the following time points: Five days before the operation, intraoperatively directly after liver resection and on POD 3, 7 and 15, respectively. LiMAx was performed after a minimal fasting time of 8 h. For the preoperative test, pigs were sedated with an intramuscular injection of 5 mg/kg tiletamine, 5 mg/kg zolazepam and 0.04–0.08 mg/kg atropine. The postoperative tests were performed under sedation with repeated intravenous application of propofol (1–5 mg/kg). Before injection of ^13^C-methacetin, the baseline ^13^CO_2_/^12^CO_2_ ratio was ascertained in the native expiration. An individual baseline was set for the delta-over-base calculation ^13^CO_2_/^12^CO_2_ values. After intravenous administration of 2 mg/kg ^13^C-methacetin (^13^C-Methacetin-Solution, Humedics GmbH) as previously described [22], followed by 20 ml of 0.9% sodium chloride, the ^13^CO_2_/^12^CO_2_ ratio dynamic was measured over a time period of at least 30 min and the LiMAx value was calculated following the previously described formula [22].

### Measurement of venous pressures

Before and after the resection portal venous and central venous pressures were determined invasively via the appropriate catheters described above, respectively, using a common clinical pressure transducer (DPT-6000, Codan pvb Critical Care GmbH, Forstinning, Germany).

### Statistical analysis

Statistical analysis was performed using GraphPad PRISM 5. The *Mann-Whitney-U test*, *Wilcoxon matched pairs test* and *Pearson correlation coefficient test* were applied as appropriate. Differences were regarded statistically significant with p < 0.05. Results were expressed as mean ± SEM unless otherwise indicated. In order to calculate the liver weight of LEWE minipigs, first testing for normal distribution (*Kurtosis and Skewness test*) was performed. Thereafter logistic regression analysis between calculated liver weight as a dependent variable and body weight was tested. ANOVA Test was applied for testing regression significance.

## Results

### Preoperative preparation for the surgical procedure

The preoperative prokinetic therapy was well tolerated by all animals. Especially during the fasting period of 24 hours prior to surgery animals were visited frequently and showed excellent clinical behavior. None of the animals showed vomiting, foetor ex ore or clinical symptoms of hypoglycemia. The preoperative blood gas analyses showed normal pH and glucose levels (7.44 ± 0.01 and 3.8 ± 0.3 mmol/l, respectively).

### Estimation of liver weight before resection

Following 13 total hepatectomies in LEWE minipigs, a mean liver weight of 605.9 ± 31.7 g and a mean body weight of 44.4 ± 2.6 kg were obtained. Logistic regression was performed to estimate the liver weight before resection in our experimental group and resulted in the following equation to calculate liver weight in our LEWE minipigs: LW = 491.86 *ln(BW)– 1249.6 (R^2^ = 0.807) (Fig 2).

### Surgical procedure and perioperative courses

In 4 animals, left trisegmentectomy was performed and in 3 animals, additional partial right lateral sectionectomy was carried out in order to achieve a FLR of approximately 50%. Mean mass of resected liver parenchyma was 322.0 ± 10.2 g, representing 48.8 ± 3.2% of estimated total liver mass. According to pulse oximetry, repetitive non-invasive blood pressure measurement and capnometry, all animals remained cardiopulmonary stable throughout the entire operation. Intraoperatively, a mean amount of 2571 ± 105 ml isotonic electrolyte solution was applied and there were no needs for vasopressors or blood transfusion. Overall, animals on average received 5257 ± 356 ml isotonic electrolytic solution within the first 24 hours after the start of the operation.

**Fig 4:**
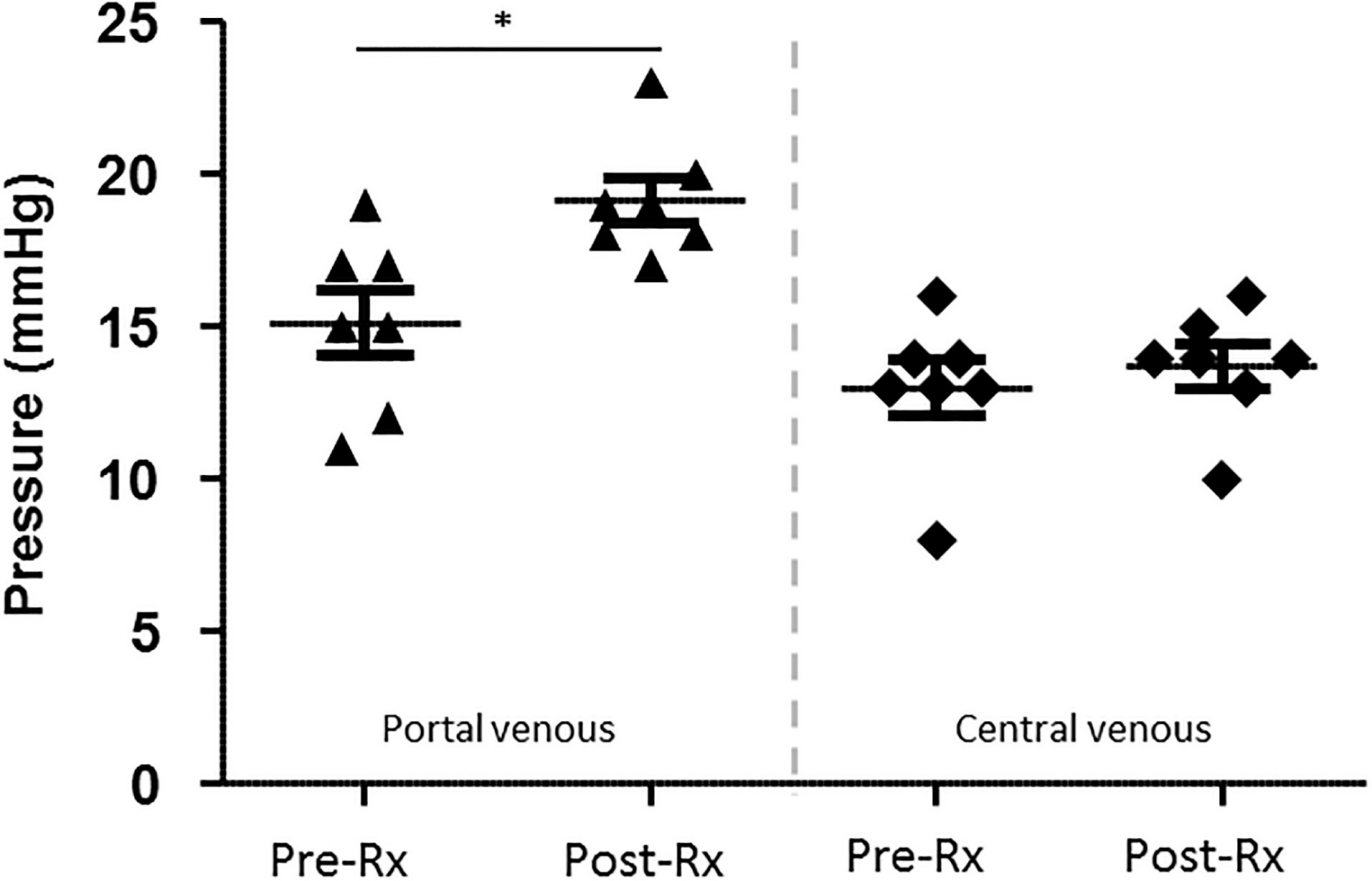
Comparison of portal and central venous pressures. Scatter plot depicting the portal venous (black arrows) and central venous (black squares) pressures before and after liver resection determined via the appropriate catheters. (Pre-Rx: Prior to liver resection; Post-Rx: After liver resection; data presented with mean ± standard error of mean; n = 7, * p < 0.05).

Mean preoperative central venous pressure (CVP) was 13.0 ± 0.9 mmHg, mean preoperative portal venous pressure (PVP) was 15.1 ± 1.1 mmHg. Corresponding postoperative values showed increases to 13.7 ± 0.7 mmHg (+5.5%, p = 0.173) and 19.1 ± 0.7 mmHg (+26.4%, p = 0.022), respectively (Fig 4).

**Fig 5:**
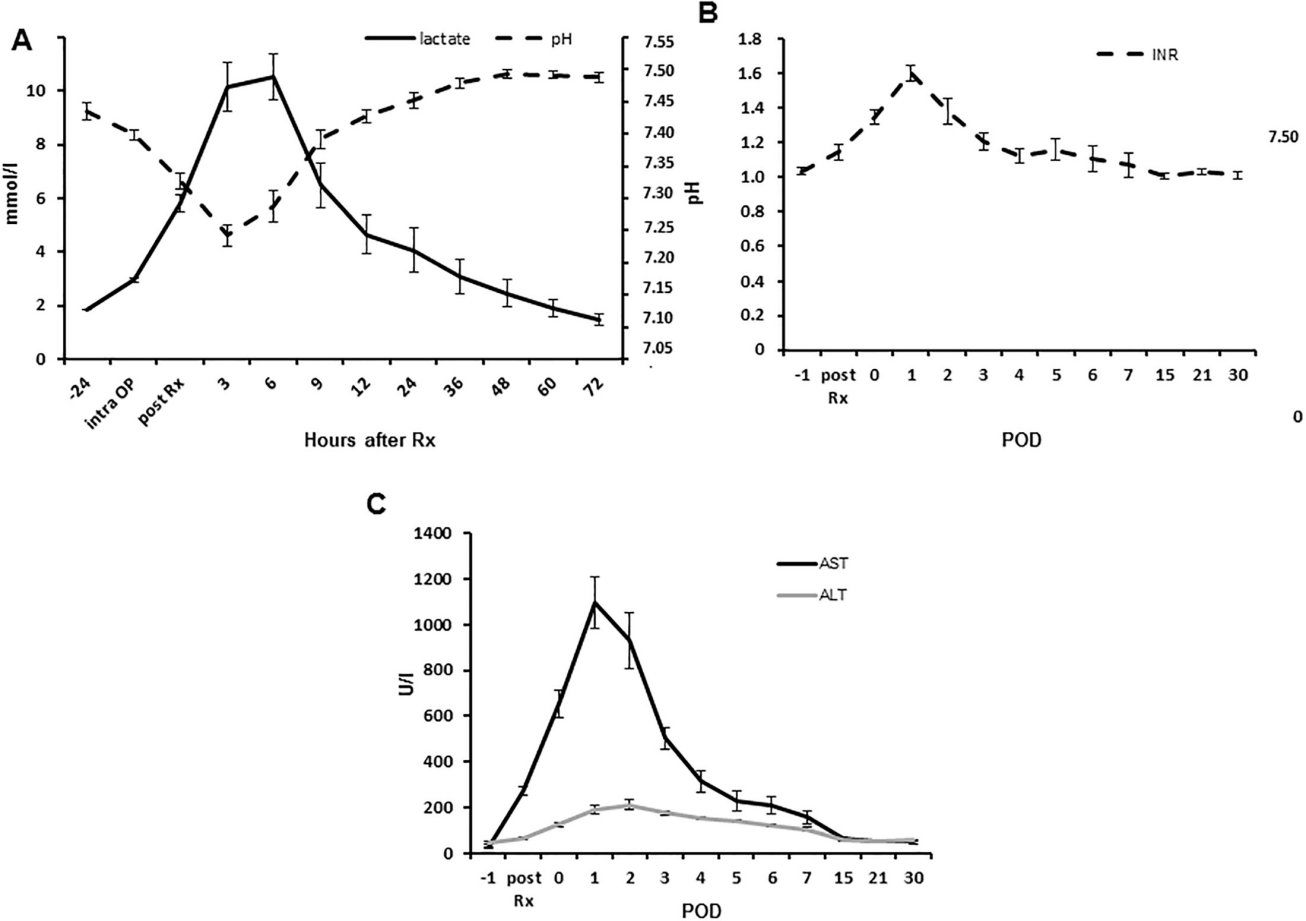
Postoperative course of laboratory findings. (**A**) Diagram depicting the courses of lactate (mmol/l; solid black line) and pH (dotted black line) determined in the blood gas analyses following major liver resection. (**B**) Diagram depicting course of INR (dotted black line) following major liver resection. (**C**) Diagram depicting the courses of AST (mmol/l; solid black line) and ALT (mmol/l; solid grey line) following major liver resection, respectively (Rx: Liver resection; POD: Postoperative day; Data presented as mean ± standard error of mean; n = 7).

Mean preoperative hemoglobin (Hb) was 12.2 ± 0.4 g/dl with hematocrit (Hct) of 37.7 ± 1.2% and lactate at 1.8 ± 0.3 mmol/l. Six hours after surgery the values of Hb and Hct decreased to 12.0 ± 0.4 g/dl (p = 0.498) and 37.0 ± 1.3% (p = 0.6875), respectively, meanwhile lactate showed an increase to 10.5 ± 0.9 mmol/l (p = 0.016 all compared to baseline). However, lactate stabilized upon fluid therapy during POD 1, quickly reaching baseline levels (Fig 5A); simultaneously also Hb and Hct normalized with no need of blood transfusions (S1 Fig).

Six hours after resection, the INR value and coagulation factor II were already significantly deranged compared to preoperative values (INR: 1.03 ± 0.1 vs. 1.3 ± 0.1 with p = 0.003 and coagulation factor II: 69.8 ± 2.2% vs. 44.3 ±. 2.3% with p = 0.02 for pre- and postoperative measurements, respectively; Fig 5B). For both parameters, the largest discrepancy to baseline values were obtained on POD 1 (INR: 1.6 ± 0.1, p = 0.003; coagulation factor II: 38.1 ± 2.6%, p = 0.006), followed by a trend to normalization on POD 2 (INR: 1.4 ± 0.1; coagulation factor II: 38.4 ± 3.0%; with p = 0.0781 and 0.9325 compared to POD1, respectively) and a return to the initial value range on POD 7 and 6, respectively.

A similar dynamic was obtained for AST and ALT levels which raised directly after the operation and peaked on POD 1 (AST) and POD 2 (ALT), respectively, as shown in Fig 5B. Maximum values of 1096 ± 119.7 U/l and 210.6 ± 22.9 U/l were observed (p = 0.002 and 0.002 to baseline), respectively.

**Fig 6:**
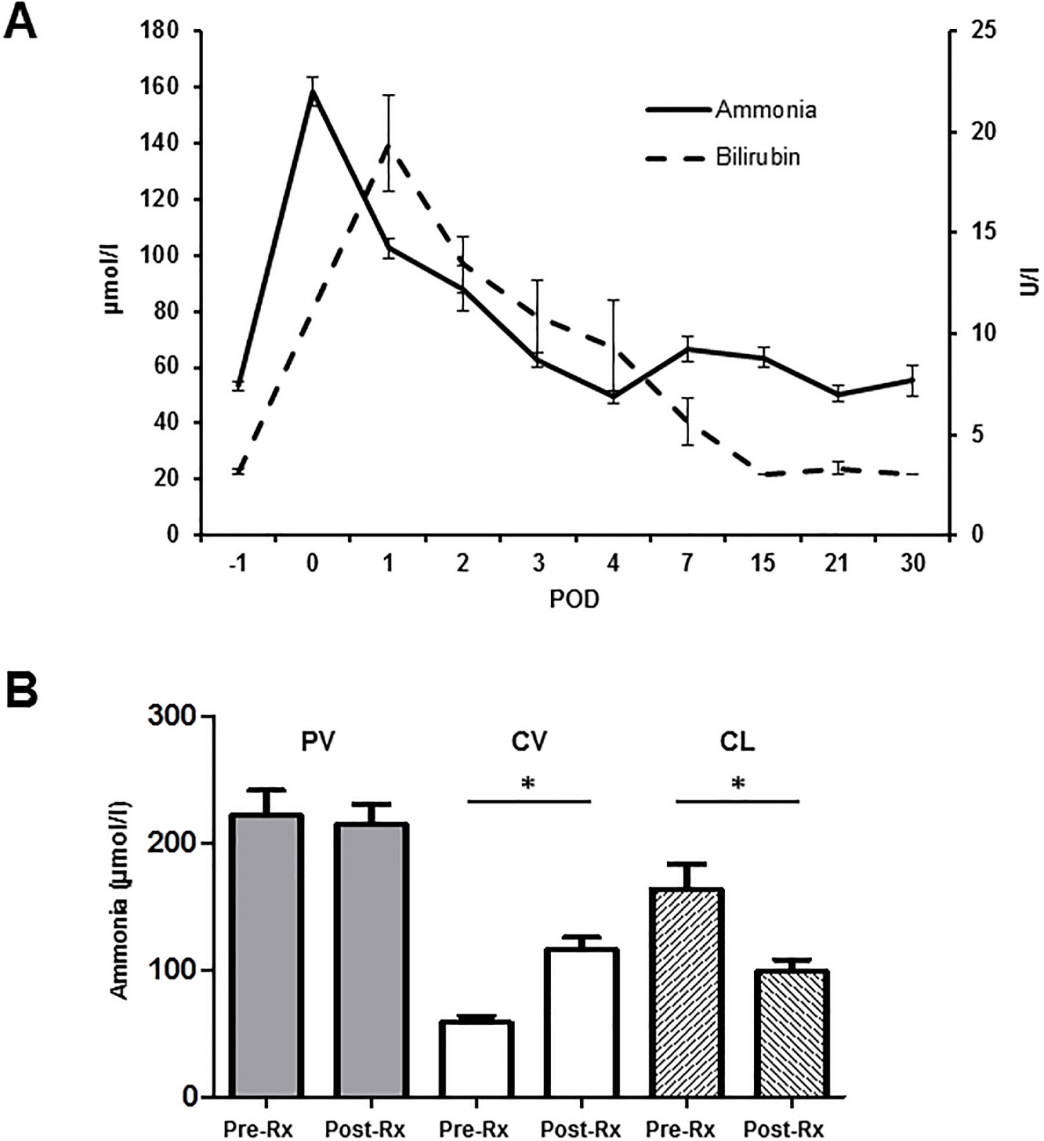
Development of ammonia and bilirubin levels after major liver resection. (**A**) Diagram depicting the courses of ammonia (solid black line) and bilirubin (dotted black line) following major liver resection. (**B**) Bar chart depicting serum levels of ammonia before and after liver resection determined in the blood withdrawn from the portal vein (PV, grey bars) and the central vein (CV, white bars). Furthermore, the calculated ammonia clearance (CL, striped bars) before and after resection is shown. (POD: Postoperative day; Pre-Rx: Prior to liver resection; Post-Rx: After liver resection; Data presented as mean ± standard error of mean; n = 7, * p < 0.05).

The mean total bilirubin peaked with a value of 19.4 ± 2.3 μmol/l (p = 0.001 to baseline) shortly after resection, but remained within human reference intervals as shown in Fig 6A. Levels of GGT raised from 90.7 ± 8.3 U/l to a maximum of 117.0 ± 33.5 U/l (p = 0.643 to baseline) on POD 15. The pancreas was mobilized in order to allow for implantation of the portal vein catheter. In consequence, serum lipase levels raised from 13.6 ± 2.3 U/l to 45.0 ± 7.9 U/L (p = 0.016 to baseline) on POD 2 after the resection, but remained within human reference intervals. The levels of serum amylase were not significantly affected by the surgical procedure (S2 Fig).

The central venous ammonia levels peaked at a mean of 158.3 ± 17.1 (baseline at 53.4 ± 1.5; p = 0.002) already 6 hours after liver resection and continuously dropped thereafter again reaching baseline at POD 3 to 4 (Fig 6A). Mean ammonia clearance before major liver resection was 163.4 ± 20.1 μmol/l, following resection the liver remnant was only able to reduce the ammonia levels by 98.8 ± 9.3 μmol/l as shown in Fig 6B (p = 0.015). No significant differences in portal venous ammonia levels prior to and after liver resection were observed.

### Maximum liver function capacity (LiMAx)

**Fig 7:**
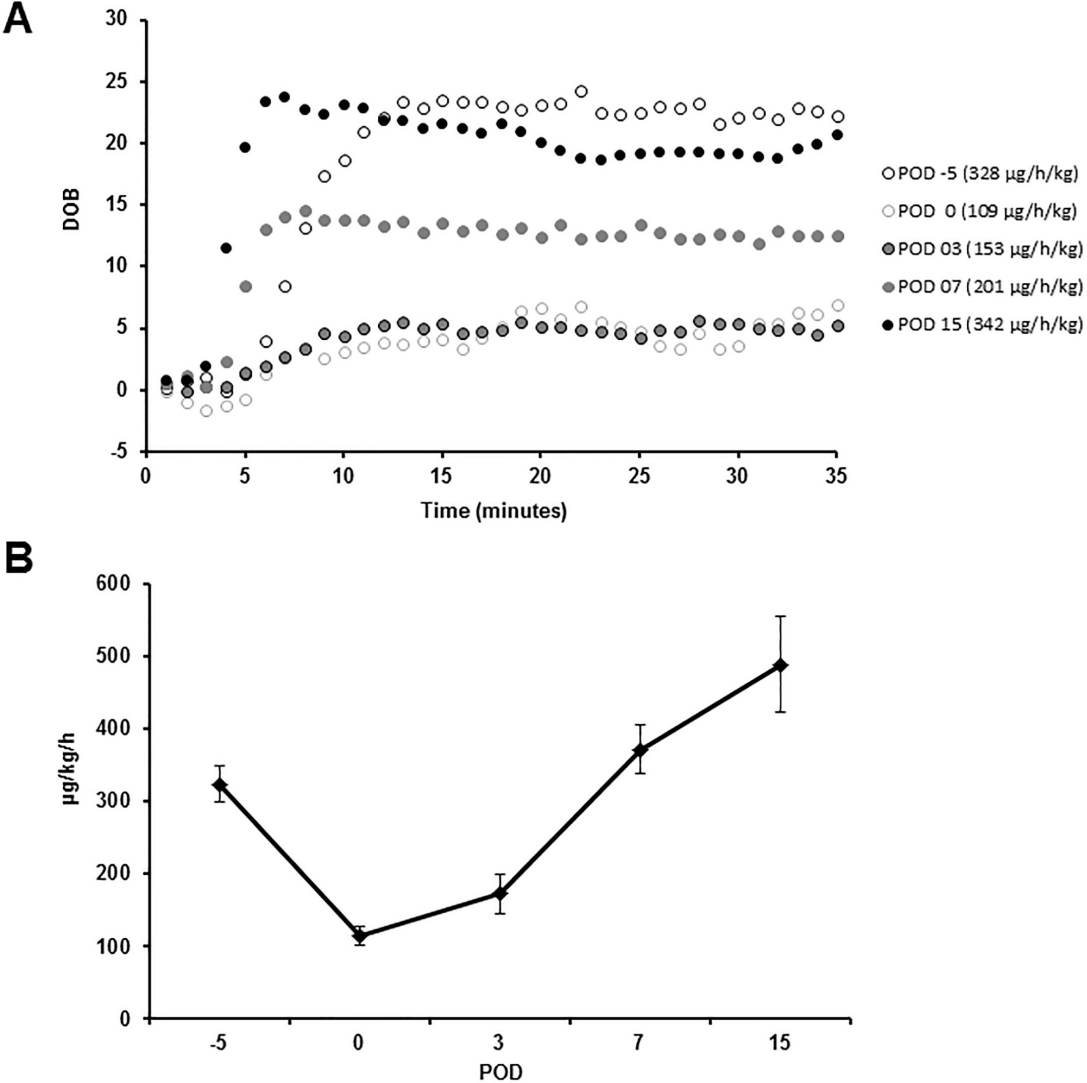
Development of maximum liver function capacity after liver resection. (**A**) Diagram depicting the development of the maximum liver function capacity (LiMAx) on POD -5, 0, 3, 7 and 15 of liver resection for one representative subject (DOB: Delta over baseline; time-dependent changes in ^13^CO_2_/^12^CO_2_-ratio given in per mil (‰)). The LiMAx device (Flip 1.0) determined the DOB in one-minute-intervals, so results of measurements are illustrated as dotted lines. The individual LiMAx values are presented in brackets. (**B**) Diagram summarizing the course of the LiMAx value (μg/kg/h) prior to and after major liver resection for all animals. (POD: Postoperative day; Data presented as mean ± standard error of mean; n = 7).

Mean preoperative LiMAx value was 324.0 ± 25.1 μg/kg/h. Immediately after liver resection (still intraoperatively) the value decreased to 114.7 ± 12.8 μg/kg/h (p = 0.001 to baseline) and showed only a slight increase to 173.0 ± 27.2.9 μg/kg/h on POD 3 (p = 0.005 to baseline). On POD 7, the LiMAx value exceeded baseline and was found at 372.2 ± 36.2 μg/kg/h (p = 0.234 to baseline) and increased even further to 489.3 ± 73.0 μg/kg/h (p = 0.073 to baseline) on POD 15 (Fig 7B). It must be noted that in 2 animals LiMAx values on POD 0 (intraoperatively) were below 80 μg/kg/h, indicating distinct liver function impairment. The LiMAx value showed a significant negative correlation with the INR (p = 0.0063; r = -0.49; S3 Fig) and the bilirubin (p < 0.0001; r = -0.65; S3B Fig). However, the LiMAx test could indicate liver function impairment 1 day earlier than conventional laboratory parameters such as INR and bilirubin (lowest LiMAx value on POD 0.14 compared to lowest INR and bilirubin values on POD 1.14, p = 0.0027).

### Morbidity and mortality

Five animals showed elevated body temperature up to 39.8 °C in-between POD 4 to 11, but without any reduction in clinical behavior or food intake. In 3 animals rectal body temperature turned back to reference value within 24 h; in 2 animal’s body temperature was elevated over a period of seven days. Furthermore, a particularly reduced bowel function over the first few days after surgery was found in most animals in terms of a postoperative intestinal motility disturbance. In this context clinical signs of a paralytic ileus were observed on POD 4 and 7, respectively, in 2 of 7 animals with reduced food intake and defecation, and finally vomitus. After 48 h of laxative measures bowel function returned and both animals showed good food intake.

One of 7 animals reached humane end points requiring euthanasia prior to scheduled end of experiment (POD 30), presenting with clinical signs of hepatic encephalopathy on POD 3 and continuously increasing ammonia levels of 135 μmol/l and 189 μmol/l on POD 1 and 2, respectively. Therefore the mortality in this cohort was 14.3%.

## Discussion

The liver has the unique ability for regeneration within a short time period after partial hepatectomy [34, 35]. In humans, this process starts within the first day after a major resection and has led to the development of novel strategies in liver surgery such as two-stage hepatectomy, portal vein embolization/ligation or ALPPS (Associating Liver Partition and Portal Vein Ligation for Staged Hepatectomy) [36]. The understanding of liver regeneration is essential for many patients with liver tumors since a curative therapy is often only possible performing extended liver resection. We therefore developed a standardized partial hepatectomy model in the miniature pig to study the underlying mechanism of liver regeneration with the help of the LiMAx test as a non-invasive liver function diagnostic and a permanent portal venous catheter.

Our protocol was associated with postoperative symptoms of reduced plasmatic coagulation, raised bilirubin and increase of portal venous pressure with no special need for postoperative intensive care therapy apart from adjusted fluid substitution, perioperative application of tranexamic acid and postoperative laxative therapy. Therefore, our results are essentially comparable to patients experiencing low grade post hepatectomy liver failure (PHLF) according to the ISGLS classification [5].

The aim of our established animal model was to generate all symptoms of decreased liver function following major liver resection without the risk of high mortality close to the clinical reality. There are several other studies of partial liver resection and consequent PHLF [37–40]. In contrast to our study, most of these published models recommend a FLR of only 10–35% which results in high a mortality [37, 38]. Especially the resection of 90% of the liver parenchyma will surely result in fatal liver failure rather than establishment of a regenerative milieu. The comparison of all these models is difficult, since various resection techniques have been applied in different pig breeds. Furthermore, the estimation of the FLR depends on different assumptions of the volume of the left three liver lobes, which are estimated between 70–80% in many of the experiments [40, 41]. The calculation of the FLR in our protocol was based on the results of a logistic regression model estimated from total hepatectomies in pigs of the same breed. Furthermore, in most experiments only a subtotal resection of the three left liver lobes is performed with a relatively large central remnant due to the special anatomy of the pig`s liver veins and vena cava which run intrahepatic. Therefore, in our opinion the extent of resection often is overestimated. Golriz et al. published a porcine model of liver resection with 3 groups with a remnant liver volume of 50%, 25% and 15%, respectively [37]. They conclude a FLR of 25% in a porcine model would be ideal to study liver regeneration despite the fact that only 1 animal survived until the end of the experiment (8 days), meanwhile animals with 50% FLR showed a low mortality but no significant increase of INR and bilirubin. In our model, we were able to show a very low mortality rate but also a significant reduction of liver function in order to analyze consecutive liver regeneration. The LiMAx test confirmed a significant reduction of liver function on POD 0 and 3, respectively, with again normalized values one week after the resection. Results were in line with obtained dynamics of laboratory parameters such as INR and coagulation factor II. In a recent study, Jara et al. showed that in healthy patients the normal range of LiMAx was 430 ± 86 μg/kg/h, therefore a mean of 324.0 ± 27.1 μg/kg/h seems a comparable value bearing in mind that the mean weight of our pigs was only 49.9 ± 5.3 kg [42]. On POD 3, the mean LiMAx was found at 114.7 ± 12.8 μg/h/kg, representing 56% of the preoperative value which still nearly matches the estimated extent of liver resection performed in our pigs, despite regeneration already has taken place for a few days. This indicates that the degree of liver resection correlates with the measured LiMAx values. From our knowledge, this is the first time that the LiMAx test was performed in a chronic porcine model of liver resection, and according to our data, LiMAx appeared capable to asses liver function non-invasively as described for humans. The LiMAx test enables real time/live monitoring of the liver’s metabolism in comparison to the conventional laboratory parameters such as INR and bilirubin that are the result of processes already taken place. In this study, the LiMAx test already showed significant liver function impairment shortly after liver resection (0.14 days) while similar impairment of INR and bilirubin could only be detected 24 hours later (1.14 days). In the animal setting this test further has the advantage of being less logistically challenging compared to determination of liver function by radiological imaging (e.g. volumetry of liver by CT/MRI or ^99^mTc-mebrofenin hepatobiliary scintigraphy with SPECT-CT).

Another obvious advantage of the presented model is the possibility to steadily measure laboratory parameters as well as central venous and portal venous pressures without the need for sedation or additional puncture of the central or portal vein. There are different approaches of portal vein catheterization in pigs and it was first described in detail by Lydtin et al. [43] in 1969. They developed a technique in which the portal venous catheter was introduced into the mesenteric vein of the ileocecal junction. Recently Kaiser et al. published a series of portal venous catheters inserted through the pancreatic vein into the portal vein within the hepatoduodenal ligament [44]. Our technique is similar to the procedure published by Van Leuwen [45] including vascular clamping of the portal vein prior to incision and fixation of the catheter by suturing. Overall, the presented technique to implant the portal venous catheter is well reproducible, safe, easy to perform and the obvious risk of pancreatic injury due the close anatomical position was not evident in any of the animals.

Apart from measuring the portal venous pressure, which has been proposed as an independent factor of PHLF with increase of 90 day-mortality by Allard et al. [46], a portal venous catheter has the advantage of comparing blood samples taken from the central and the portal venous blood stream which amongst others is of special interest for the study of pharmacokinetics and also the first-pass effect of drugs [47]. Furthermore, the measurement of portal venous ammonia in comparison with central venous ammonia, as we presented in this study, could represent an additional diagnostic measure concerning determination of real time liver function intraoperatively.

In general, the postoperative morbidity and mortality in this study concerning the portal and central venous catheter as well as the liver resection was low. Postoperative ileus is a known complication after abdominal surgery in pig models [48]. In earlier studies, we also faced this complication frequently prior to implementation of a preoperative diet as well as a perioperative prokinetic therapy into our treatment protocol. In the present study, we could still observe symptoms of postoperative intestinal motility disturbance in some pigs, but no serious events with a need for surgical revision or euthanasia.

In conclusion, the presented porcine model of major liver resection proved suitable to study liver regeneration with low morbidity and mortality and indicated that the LiMAx test can be applied in pigs. Implantation as well as long-term maintenance of a permanent portal vein catheter in this setting was feasible without relevant complications and allowed repetitive measurement of portal venous pressure as well as steady vascular access.

## Supporting information

S1 Fig Development of hemoglobin and hematocrit after major liver resection. Diagram depicting the courses of hemoglobin (g/dl; solid black line; Hb) and hematocrit (%; dotted black line, Hct) determined in the blood gas analyses in the early phase after major liver resection.
(TIF)

S2 Fig Development of serum amylase and lipase after major liver resection. Diagram depicting the courses of amylase (U/l; solid black line) and Lipase (U/l; dotted black line) determined as part of the laboratory profile following major liver resection.
(TIF)

S3 Fig Correlation between LiMAx value and conventional laboratory parameters of liver function. Diagrams depicting the correlation between the LiMAx value and the appropriate INR (**A**) and bilirubin (**B**) values, respectively, determined at the same time point in the course after major liver resection. Data presented with linear regression curves (solid black lines; INR = -0.0004*LiMAx + 1.2092 (R^2^ = 0.24492); Bilirubin = -0.0292*LiMAx + 17.045) (R^2^ = 0.4038)).
(TIF)
