# Supplementary figures and images for: Porcine model for the study of liver regeneration enhanced by non-invasive 13C-methacetin breath test (LiMAx test) and permanent portal venous access
Source: PLoS One. 2019 May 31;14(5):e0217488. doi: 10.1371/journal.pone.0217488 (PMC6544243; doi:10.1371/journal.pone.0217488)

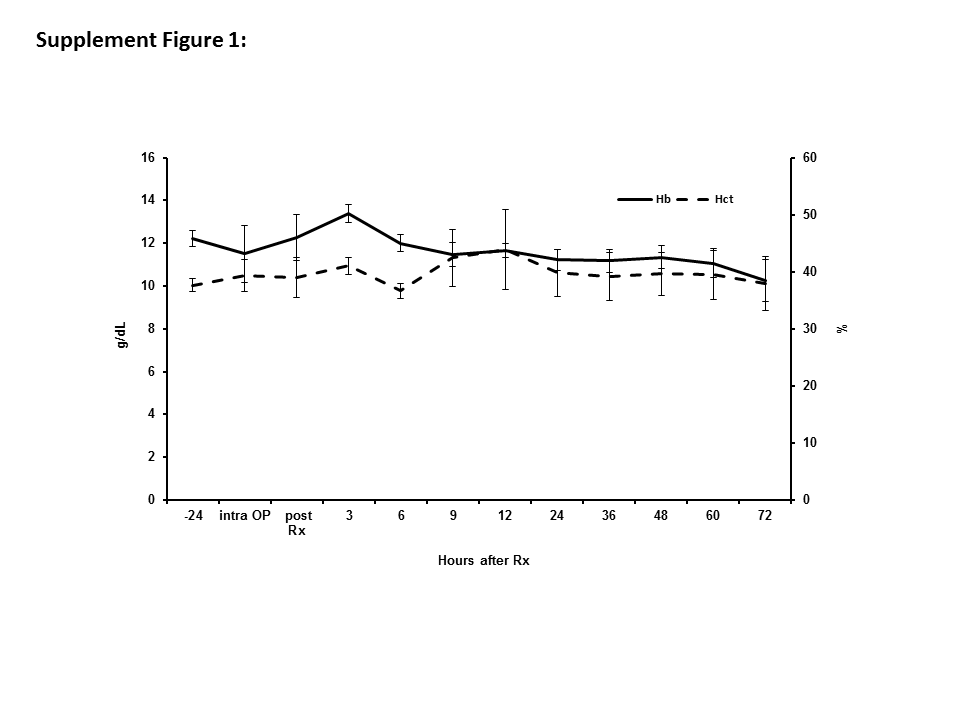

Supplement: S1 Fig — Diagram depicting the courses of hemoglobin (g/dl; solid black line; Hb) and hematocrit (%; dotted black line, Hct) determined in the blood gas analyses in the early phase after major liver resection. (TIF) [file pone.0217488.s001.tif]

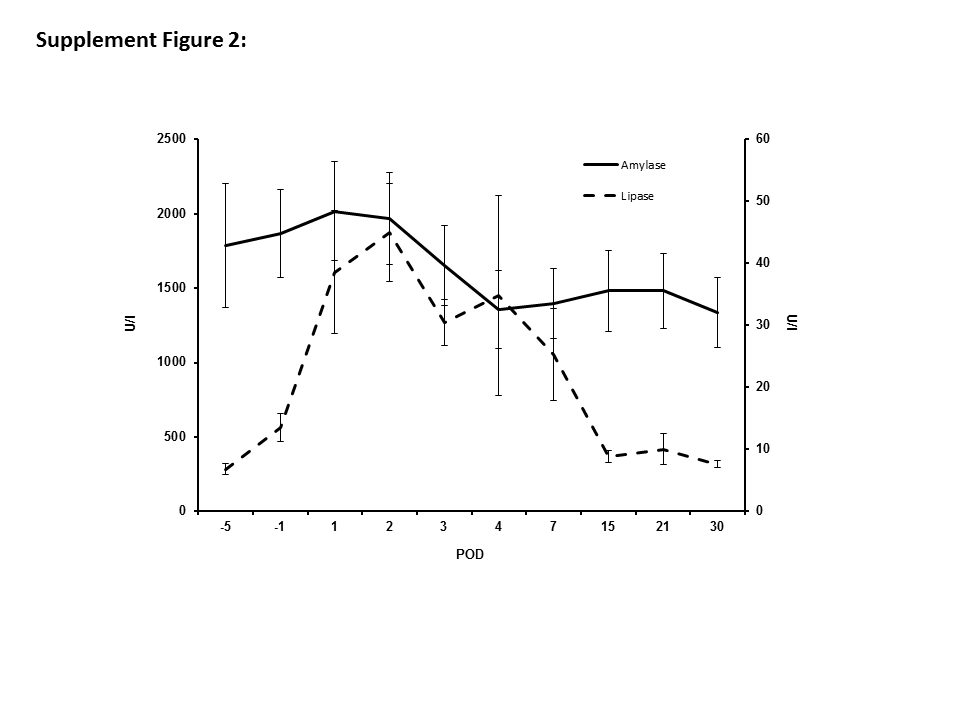

Supplement: S2 Fig — Diagram depicting the courses of amylase (U/l; solid black line) and Lipase (U/l; dotted black line) determined as part of the laboratory profile following major liver resection. (TIF) [file pone.0217488.s002.tif]

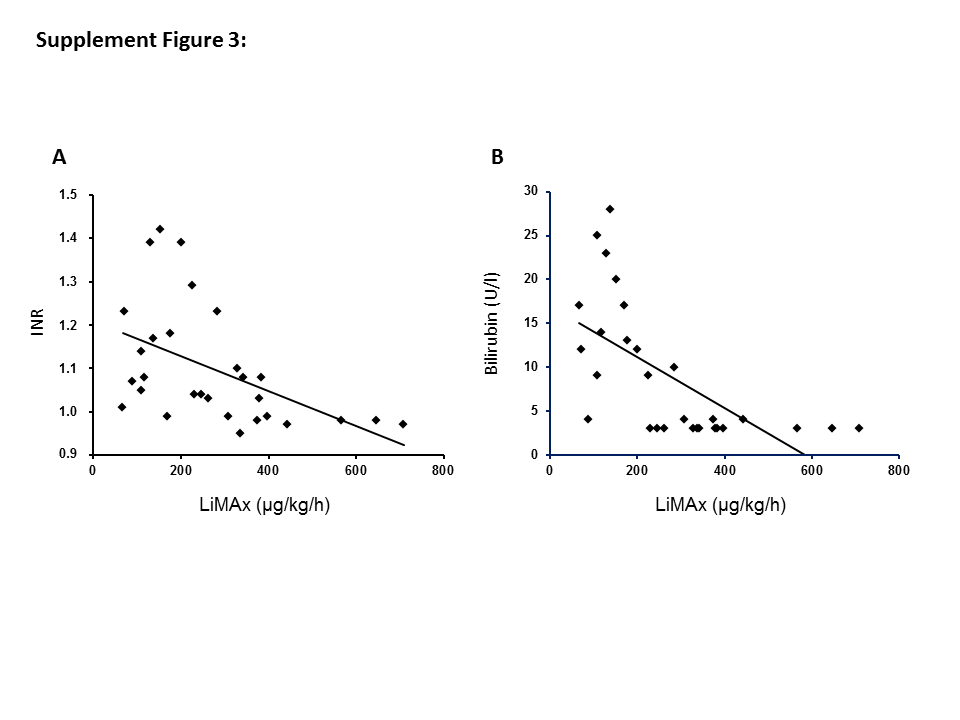

Supplement: S3 Fig — Diagrams depicting the correlation between the LiMAx value and the appropriate INR (A) and bilirubin (B) values, respectively, determined at the same time point in the course after major liver resection. Data presented with linear regression curves (solid black lines; INR = -0.0004*LiMAx + 1.2092 (R2 = 0.24492); Bilirubin = -0.0292*LiMAx + 17.045) (R2 = 0.4038)). (TIF) [file pone.0217488.s003.tif]
